# The Kinematics of Scale Deflection in the Course of Multi-Step Seed Extraction from European Larch Cones (*Larix decidua* Mill.) Taking into Account Their Cellular Structure

**DOI:** 10.3390/ma14174913

**Published:** 2021-08-29

**Authors:** Ewa Tulska, Monika Aniszewska, Arkadiusz Gendek

**Affiliations:** Department of Biosystems Engineering, Institute of Mechanical Engineering, Warsaw University of Life Sciences, Nowoursynowska 164, 02-787 Warsaw, Poland; ewa_tulska@sggw.edu.pl (E.T.); monika_aniszewska@sggw.edu.pl (M.A.)

**Keywords:** scale opening mechanics, seed extraction, morphological structure

## Abstract

The objective of the study was to elucidate the kinematics of cone opening in the European larch (*Larix decidua* Mill.) during a four-step seed extraction process and to determine optimum process time on that basis. Each step lasted 8 h with 10 min of water immersion between the steps. The study also described the microscopic cellular structure of scales in cones with a moisture content of 5% and 20%, as well as evaluated changes in cell wall thickness. The obtained results were compared with the structural investigations of scales conducted using scanning electron microscopy (SEM) of characteristic sites on the inner and outer sides of the scales. The greatest increment in the scale opening angle was noted on the first day of the process (34°) and in scales from the middle cone segment (39°). In scales with a moisture content of 5% and 20%, the greatest changes in cell wall thickness were recorded for large cells (57%). The inner and outer structure of scales differed in terms of the presence and size of cells depending on the moisture content of the cones (5%, 10%, or 20%). The study demonstrated that the moisture content of cones was the crucial determinant of the cellular structure and opening of scales in larch cones. The scale opening angle increased with decreasing moisture content but did not differ significantly for various segments of cones or various hours of the consecutive days of the process. This finding may lead to reducing the seed extraction time for larch cones. The internal and external structure of scales differed depending on moisture content, which also determined the size and wall thickness of cells.

## 1. Introduction

Seed extraction from the cones of various forest tree species has been described in the research literature since the 1950s [1,2] as a complex process [3] determined by taxonomic characteristics [4]. Most publications on the subject tend to analyze pine seed extraction due to the fact that the seeds of that species are in greatest demand [5,6,7]. Conifer seeds constitute a valuable propagation material needed for forest regeneration either via natural processes or for the needs of nurseries [8].

On average, between 200 and 500 kg of cones and 17.5 to 28.0 kg of seeds can be harvested per 1 ha of larch stands in Poland [9,10]. In Polish conditions, approximately 10,000 kg of larch cones were harvested annually between 2010 and 2020. In years of low harvest, there is a significant proportion of empty cones, which may be attributed to pests or diseases [11], or even climate change [12,13]. Furthermore, the total area of larch stands in Poland is to be reduced by 20% [9]. In view of these factors, it seems important to add to the understanding of the process of seed extraction from larch cones to maximize the amount and quality of seeds and enable their long-term storage.

Post-extraction cones constitute waste, which can be briquetted [14] or torrefied [15] and used together with damaged seeds for power generation purposes [16,17,18].

In practice, seeds are obtained from larch cones in two ways: via thermal extraction (involving alternating drying and moistening of cones) or thermal-mechanical extraction (long-term drying with additional mechanical crushing of scales) [19,20,21]. In the first method, moistening treatments extend the seed extraction time considerably, up to 60 h [22]. In addition, seed shaking in devices manufactured by BCC (Sweden), Nomeko (Sweden), or OTL Jarocin (Poland) is carried out between drying stages, directly before moistening [23,24,25]. Seeds obtained by thermal extraction are easier to clean, and it is possible to obtain nearly 100% purity. The second method, in turn, carries the risk of damaging the coat of the obtained seeds by the grinding elements of cone crushing equipment [20]. As reported by Suszka [21], mechanical extraction of seeds from larch cones was attempted by Drachal and Tyszkiewicz using a self-developed device, TD Mechanical Seed Extractor. The separation of seeds from a mixture of dust and cone debris makes the method difficult to implement [19].

In Poland, seeds from larch cones are extracted using pine and spruce extraction programs in seed extraction cabinets using two-step extraction programs with variable drying temperature [26] to prevent thermal damage to the seeds [1]. Researchers seek new devices and technological solutions to make the process more effective, for example, by microwave irradiation of cones in the initial stage of seed extraction [27,28].

The structure and properties of cell layers may affect the mechanical movement of scales [29]. The humidity of air surrounding the cones has a significant impact on moisture absorption and transpiration of the water vapor contained in the scale cells, which, due to changes in the temperature of the drying air, expand and contract anisotropically in a direction perpendicular to scale tissue orientation [30,31]. Periodic changes in the moisture content of larch cones after reaching the preliminary dry state lead to the contraction and relaxation of scale cells, causing scale movement and outward displacement of the seeds [20]. The process is gradual, and the seeds are released from the cones only after several instances of cone opening and closing [19]. Under natural conditions, approx. three weeks after the beginning of spring the upper parts of seed wings begin to project by approx. 2–3 mm outside the scales in cones on trees. Subsequently, following a slight decrease in cone moisture the scales are gradually deflected and the seeds fall out. Partially displaced seeds do not slide back to their initial positions, even after cone moistening. This is due to the fact that the space under the scale is the narrowest at the cone rachis (where the seed was originally located) and becomes wider in the outward direction [20]. Specific mechanisms of scale opening and closing are linked to plant evolution and survival strategy, which enables conifers to release seeds to greater distances on sunny and dry days [32].

In addition to a publication by Aniszewska [33,34], the available literature provides some other studies on the cone structure and the scale opening process [29,35,36,37], but these do not concern European larch cones.

The research problem addressed in this paper concerns difficulties with seed extraction from larch cones associated with their scale structure. Thus, the study evaluates the kinematics of scale deflection caused by changes in moisture content in the cones, the cellular structure of scales, and the resulting changes in cell wall thickness during scale opening. It also examines the scale surface in the process of seed extraction.

## 2. Materials and Methods

### 2.1. Provenance and Characterization of the Material

The study involved European larch cones (MP/3/41001/05) collected at the beginning of December 2019 from the seed orchard at the Grabowiec Nursery, division 282 k, Bielsk Podlaski municipality, Podlaskie Province (GPS: 52°41′0 N, 23°60′ E). The cones were transferred to the laboratory of the Department of Biosystems Engineering, Warsaw University of Life Sciences; divided into batches; and stored in an LKexv 3600 laboratory refrigerator (Liebherr, Bulle, Switzerland) at 2 ± 1 °C until examination. The length and thickness of all cones were measured (length—*h* and diameter—*d*) using a Silverline 677,256 electronic Vernier caliper (Silverline Tools, Yeovil, UK) with an accuracy of ±0.1 mm; their initial weight m_0_ was determined using a WPS210S laboratory balance (Radwag, Radom, Poland) with an accuracy of ±0.001 g.

### 2.2. Provenance and Characterization of the Material

The mechanics of scale deflection from the rachis were examined throughout the process of seed extraction. Individual closed cones were cut in half along the axis using an originally developed blade with holder [38] mounted in a modified 10 T screw press (Cormak, Siedlce, Poland). Each cone was placed on a special base, bottom side to the baffle. Subsequently, the turn of the lever lowered the blade that cut the cone from top to bottom, perpendicularly to its axis.

**Figure 1:**
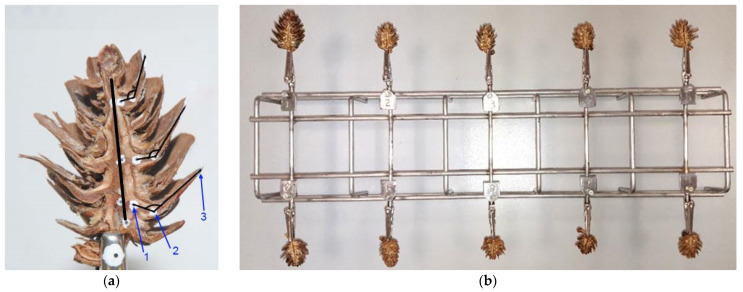
Cone half with marked reference points and axis (**a**), and stand for examining the scale opening angle in ten cone samples (**b**), where: 1—junction of the scale with the cone rachis, 2—point on the scale curve, and 3—scale apex.

In the resulting half cones, three reference points were marked on selected scales (Figure 1a): one at the junction of the scale with the cone rachis (1), another one on the curve of the scale (2), and the last one (3) at the distal end of the scale.

After marking the reference points, the prepared cone halves were placed in the holder of the purpose-developed stand to examine the opening angle of the scales (Figure 1b).

Subsequently, the cones were placed in a Heraeus UT612 circulating air oven (Kendro Laboratory Products GmbH, Hanau, Germany). The drying air temperature was set to 35 °C for the first two hours and then increased to 50 °C for another six hours. Every hour throughout the process, the stand was taken out of the oven and individual cones were photographed using a Nikon D3000 camera (Nikon, Tokyo, Japan) with an AF-S DX NIKKOR 18–105 mm f/3.5–5.6G ED VR lens. The cones on the stand were photographed against a white background with a Modeco MN 85-001 manual Vernier caliper (Modeco Expert, Wrocław, Poland), which served as a measurement reference for scaling. Images acquired at a focal length of 105 mm and an aperture of f = 5.6 were saved in JPG format at a resolution of 3872 × 2592 pixels. The distance between the cones and the lens was 350 mm.

The other half of each cone was placed on a glass disc with a diameter of *Φ* = 0.90 mm (Chemland, Stargard, Poland) in the oven next to the cone stand. After acquiring images of the first half, the other half on the glass disc was removed from the oven and weighed on WPS 210S laboratory scales (Radwag, Radom, Poland) with an accuracy of 0.001 g.

After 8 h of seed extraction and taking nine photographs of each cone half, the halves were immersed in distilled water at approx. 25 °C in laboratory beakers (Chemland, Stargard, Poland) for approx. 10 min, after which they were removed and left to soak for 14 h. The cycle was repeated over the next four days.

After the completion of seed extraction, the other halves were dried at 105 °C for 24 h to constant weight.

After the end of examination, the acquired images were analyzed using MultiScan Base v. 18.03 software (Computer Scanning System, Warsaw, Poland). In the images, three reference points on scales were connected by lines to determine the scale opening angle, α, with an accuracy of ±0.01° in each hour of the process (Figure 1a). Analysis involved scales from three cone regions: apex, middle, and base.

The methodology for investigating the scale opening angle was described by Dawson et al. [35], who studied *Pinus radiata* cones, and by Aniszewska [34], who studied *Pinus sylvestris*, *Picea abies* and *Larix deciduas* cones. It was also followed by Bae and Kim [29] in their investigation of the scale opening angle in pine cones (*Pinus*).

It was assumed that for each of the halved cones the absolute moisture content of one half mounted in a holder for photographing was the same as that of the other half on the glass disc. Therefore, moisture content in each cone was estimated on the basis of weighing its half on a glass disc and determining its dry matter content; that moisture content was then assigned to the scale opening angle at the time of measurement.

### 2.3. Cellular Structure of Cone Scales

**Figure 2:**
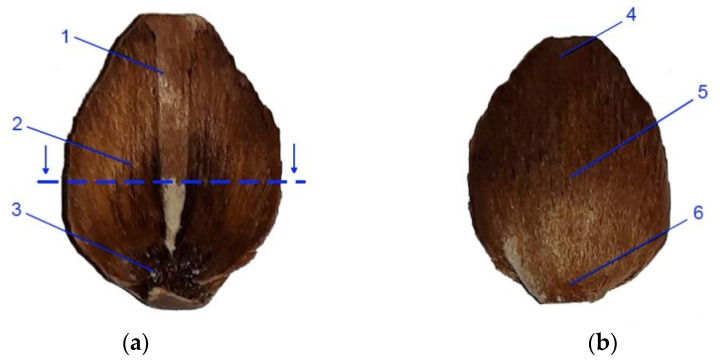
View of larch scale from the middle part of the cone: (**a**)—inner side (with the cross-section area marked): 1—wing area margin in the distal part of the scale, 2—wing area in the middle part of the scale, and 3 – seed depression in the proximal part of the scale; (**b**)—outer side: 4—distal part, 5—middle part, and 6—proximal part of the scale.

Scales for cellular structure examination were taken from the middle segment of cones with a moisture content of 5% and 20%. Cross-sections of the middle region of the scales were prepared as microscope slides (Figure 2a).

Samples of scales with a moisture content of 5% were taken using an NT Cutter BA-170 blade (NT Incorporated, Tokyo, Japan) with a WSL-lab microtome (Swiss Federal Research Institute WSL, Zürich, Switzerland). Samples of scales with a moisture content of 20% were taken using a Leica 22 C blade (Leica, Wetzlar, Germany) with a MC 2 u4.2 microtome (Moscow, Russia). The slides were observed at magnifications of ×40, ×100, and ×400. Cross-sections from scales with a moisture content of 5% were examined using an Olympus BX61 (ZEISS, Oberkohen, Germany) biological microscope coupled to an Asion 556 camera (ZEISS, Oberkohen, Germany). Cross-sections of scales with a moisture content of 20% were examined using a Nikon Alphaphot–2 YS2 biological microscope (Nikon, Tokyo, Japan) coupled to a Panasonic GP—KR222E camera (Panasonic, Kadoma, Japan). This measurement method was used for spruce cones by Aniszewska [34] and for pine cones by Bae and Kim [29].

Prior to the preparation of scale slides from cones with a moisture content of 5%, the collected scales were immersed for 15 min in plant glycerin to decrease their brittleness and enable microtome cutting; in the case of scales with a moisture content of 20%, such a treatment was not necessary.

The acquired microscopic images were analyzed by means of MultiScan Base v.18.03 (Computer Scanning System, Warsaw, Poland) and ZEN v. 2.3 software (ZEISS, Oberkohen, Germany) to measure the distance between the outer cell margin and lumen termed “cell wall thickness”, with an accuracy of 0.0001 ± µm.

### 2.4. Surface Structure of Scales under an Electron Scanning Microscope

The surface structure of scales was examined under a SEM 200 electron scanning microscope (Quanta, FEI, Europe). Scales for examination were taken from the middle region of whole cones used for cellular studies. Characteristic areas were examined both on the inner surfaces (to which seeds with wings are attached, Figure 2a) and outer surfaces of scales (Figure 2b) at magnifications of ×50 and ×500. Photographs of the inner side involved the following regions: wing area margin (1), wing area (2), and seed depression area (3), while the outer areas were the distal part of the scale (4), the middle part, adjoined by a lower scale (5), and the proximal part of the scale (6). The acquired SEM images were analyzed using MultiScan Base v. 18.03 software (Computer Scanning System, Warsaw, Poland) to measure the dimensions of the structural elements of scales with moisture contents of 5%, 10%, and 20%. The SEM-based method for determining the surface structure of scales or other plant materials is part of public domain and was described by, inter alia, Aniszewska et al. [39], Dawson et al. [35], Bae and Kim [29], and Berthlott et al. [40].

### 2.5. Statistical Analysis

The parameters were analyzed using the Statistica v.13 program (TIBCO Software Inc., Palo Alto, Santa Clara, CA, USA). Analyses of variances (ANOVA) were performed at a significance level of α = 0.05. The differences were statistically significant for *p* < 0.05.

## 3. Study Results

Table 1 presents mean values with standard deviations, as well as minimum and maximum values, ranges, and coefficients of variation for the entire set of studied cones.

The studied cones had a length of 30.0 to 33.1 mm with a mean of 31.2 ± 1.0 mm and a diameter of 15.5 to 17.1 mm with a mean of 16.3 ± 0.6 mm. The mean initial weight of the cones was 1.247 ± 0.398 g, while their mean initial dry weight was 0.946 ± 0.306 g. The number of scales per cone ranged from 45 to 61, with a mean of 53 ± 5.

### 3.1. Changes in the Scale Opening Angle during Seed Extraction from Larch Cones

**Figure 3:**
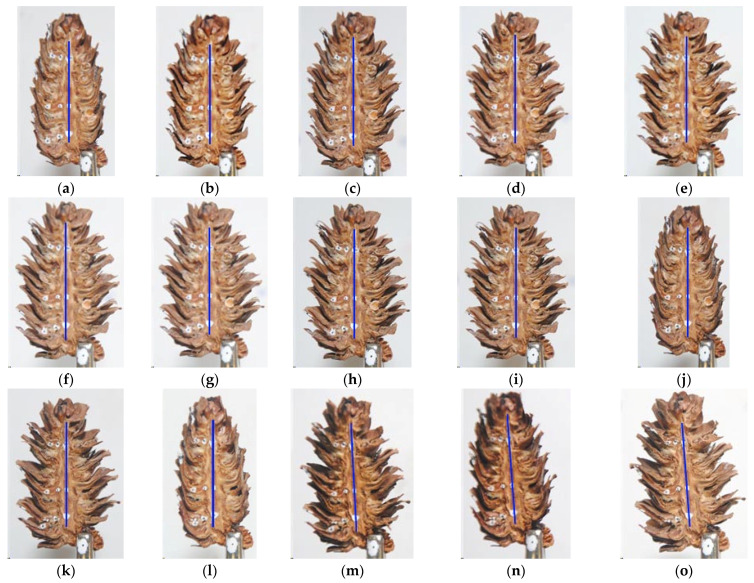
Opening states of an individual cone on the first day of seed extraction as well as at the beginning and 8 h into the process over the following days; day 1: (**a**)—initial state, (**b**)—at 1 h, (**c**)—at 2 h, (**d**)—at 3 h, (**e**)—at 4 h, (**f**)—at 5 h, (**g**)—at 6 h, (**h**)—t 7 h, and (**i**)—at 8 h; day 2: (**j**)—initial state, (**k**)—at 8 h; day 3: (**l**)—initial state, (**m**)—at 8 h, and day 4: (**n**)—initial state, and (**o**)—at 8 h.

Figure 3 presents images of the opening states of an individual cone on the first day of seed extraction, as well as at the beginning and 8 h into the process over the next days of extraction.

At the beginning of the process (day 1 initial state) the cones were fully closed; then, they gradually opened throughout the day with the greatest angles of scale deflection from the rachis being reached after 8 h. Subsequently, the cones were immersed in water for 10 min and left to absorb he moisture for 14 h. As a result, the cones closed, leading to a more intensive opening process the following day. Throughout the seed extraction process, changes in scale deflection angles were most pronounced during the first 2–3 h of extraction at the lower temperature and soon after increasing the temperature setting to 50 °C. In subsequent hours (from 4 to 8 h), the changes were imperceptible to the human eye but the opening angle continued to increase.

Table 2 shows mean moisture content values *u*_1_–*u*_4_ and scale opening angles *α*_1_–*α*_4_ together with standard deviations, measured in scales from the bottom, middle, and top cone segments for each hour of the studied seed extraction process.

The smallest mean scale opening angle at the cone base was 100.98°; it was found at the beginning of the process, at a mean cone moisture content of 0.326 ${\mathrm{kg}}_{\mathrm{water}}\cdot {\mathrm{kg}}_{\mathrm{dw}}^{-1}$. The greatest opening angle was recorded on the fourth day at 8 h; it was 136.88° at the lowest mean cone moisture content in the process (0.062 ${\mathrm{kg}}_{\mathrm{water}}\cdot {\mathrm{kg}}_{\mathrm{dw}}^{-1}$).

The mean scale opening angle at the cone base increased with each day of the process: from 100.98° to 133.88° (by 32.91°) on the first day, from 104.78° to 135.13° (by 30.35°) on the second day, from 106.79° to 135.42° (by 28.63°) on the third day, and from 111.53° to 136.88° (by 25.35°) on the fourth day.

The lowest mean scale opening angle in the middle cone segment was found at the beginning of the process (106.40°). The highest mean opening angles were recorded on the first day at 8 h (144.99°) at a mean moisture content of 0.079 ${\mathrm{kg}}_{\mathrm{water}}\cdot {\mathrm{kg}}_{\mathrm{dw}}^{-1}$ and on the second day at 8 h (144.25°) at a mean moisture content of 0.076 ${\mathrm{kg}}_{\mathrm{water}}\cdot {\mathrm{kg}}_{\mathrm{dw}}^{-1}$. At the lowest moisture content, on the fourth day at 8 h (0.062$\;{\mathrm{kg}}_{\mathrm{water}}\cdot {\mathrm{kg}}_{\mathrm{dw}}^{-1}$), the opening angle was 144.02°.

The mean scale opening angles in the middle cone segment increased from 106.40° to 144.99 (by 38.59°, the highest increment) on the first day, from 108.63° to 144.25° (by 35.63°) on the second day, from 108.37° to 143.84° (by 35.46°) on the third day, and from 113.23° to 144.02° (by 30.80°) on the fourth day.

The mean scale opening angle at the cone apex was the lowest at the beginning of the process (108.97°) and the highest on the fourth day at 8 h (140.65°). The mean initial scale opening angle at the cone apex increased with each day of seed extraction: from 108.97° to 139.14° (by 30.17°) on the first day, from 110.96° to 139.64° (by 28.68°) on the second day, from 111.09° to 139.55° (by 28.46°) on the third day, and from 114.65° to 144.65° (by 26.01°) on the fourth day.

**Figure 4:**
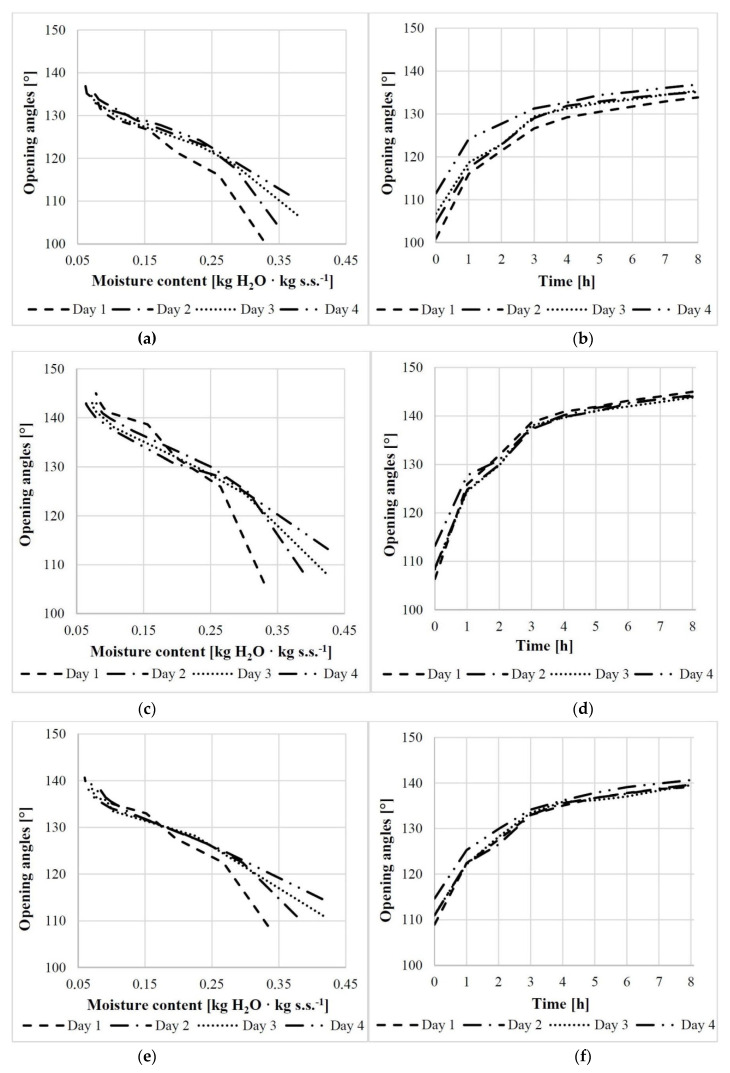
Relationship between the scale opening angle and moisture content/process duration for scales obtained from the base (**a**,**b**), middle (**c**,**d**), and apex (**e**,**f**) of cones.

The relationships between the opening angle and moisture content over the consecutive days and times are shown in Figure 4. For example, for scales from the middle cone segment the relationship was described with a polynomial Equation (1) and a linear Equation (2) for the first day and linear equations for the remaining days (3)–(5).

(1) $$\begin{array}{cc} \mathrm{Day}\;1 & \alpha_{1}=-469.53u_{1}^{2}+50.082u_{1}+142.26\;\;\;\;\;\left(R=0.991;\;t_{crit}=0.156\right), \end{array}$$

(2) $$\begin{array}{cc} \mathrm{Day}\;1 & \alpha_{1}=-131.68u_{1}+155.62\;\;\;\;\;\left(R=0.965;\;t_{crit}=0.125\right), \end{array}$$

(3) $$\begin{array}{cc} \mathrm{Day}\;2 & \alpha_{2}=-97.52u_{2}+150.92\;\;\;\;\;\left(R=0.976;\;t_{crit}=0.125\right), \end{array}$$

(4) $$\begin{array}{cc} \mathrm{Day}\;3 & \alpha_{3}\;=-91.31u_{3}+149.21\;\;\;\;\;\left(R=0.990;\;t_{crit}=0.125\right), \end{array}$$

(5) $$\begin{array}{cc} \mathrm{Day}\;4 & \alpha_{4}\;=-79.835u_{4}+147.5\;\;\;\;\;\left(R=0.992;\;t_{crit}=0.125\right), \end{array}$$

where $t_{crit}$ is the critical value of the simple or multiple correlation coefficient at α = 0.05.

For the first day, also a linear function was calculated (2) with the following opening angles: $\alpha_{oh}=112.30{}^{\circ}$, $\alpha_{1h}=120.72{}^{\circ}$, $\alpha_{2h}=129.55{}^{\circ}$, $\alpha_{3h}=135.08{}^{\circ}$, $\alpha_{4h}=142.06{}^{\circ}$, $\alpha_{5h}=143.64{}^{\circ}$, $\alpha_{6h}=143.64{}^{\circ}$, $\alpha_{7h\;}=144.82{}^{\circ}$, and $\alpha_{8h}=145.22{}^{\circ}$. The opening angle increased rapidly from the initial state up to 4 h on the first day (by 29.76°), and then slowly from 4 h to 8 h—on average by 3.16°.

The greatest increments in the mean scale opening angle at the base, middle, and apex were recorded on the first day. With increasing moisture content, the opening angle of scales in those three cone regions increased by 33.89° on the first day, 31.55° on the second day, and 30.85° on the third and fourth days.

The statistical analysis (Tukey HSD (honestly significant difference) test for unequally sized samples), which compared the scale opening angle in different sections of the cone (base, middle, and apex) for different hours on consecutive days of the process, revealed no significant differences (*p* < 0.05). This seems to indicate that the scale position in the cone does not influence the opening angle. Furthermore, the analysis demonstrated that scale opening remained similar on subsequent days, which implies that the process duration (in days) has no statistically significant impact on the scale opening angle (*p* < 0.05).

The initial moisture content of cones increased with each day, while the final moisture content continued to decrease. Moisture content changes in scales from the middle segment were 0.251 ${\mathrm{kg}}_{\mathrm{water}}\cdot {\mathrm{kg}}_{\mathrm{dw}}^{-1}$ on the first day, 0.312 ${\mathrm{kg}}_{\mathrm{water}}\cdot {\mathrm{kg}}_{\mathrm{dw}}^{-1}$ on the second day, 0.349 ${\mathrm{kg}}_{\mathrm{water}}\cdot {\mathrm{kg}}_{\mathrm{dw}}^{-1}$ on the third day, and 0.362 ${\mathrm{kg}}_{\mathrm{water}}\cdot {\mathrm{kg}}_{\mathrm{dw}}^{-1}$ on the fourth day of the process.

It was found that in scales from the middle cone segment, a decrease in moisture content of 0.01 ${\mathrm{kg}}_{\mathrm{water}}\cdot {\mathrm{kg}}_{\mathrm{dw}}^{-1}$ was associated with a mean increase in the opening angle of 1.3° on the first day and, on average, 0.90° on the following days (0.98°, 0.91°, and 0.80° on days 2, 3, and 4, respectively).

**Figure 5:**
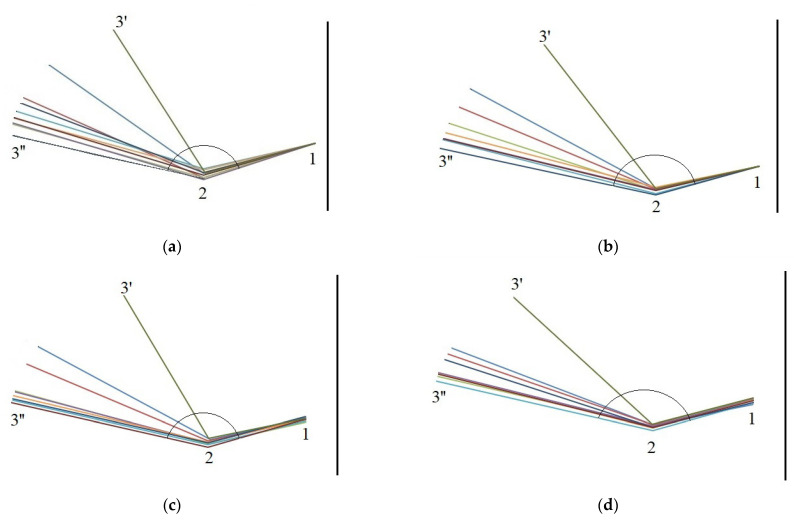
Changes in the opening angle of a scale from the middle cone segment at consecutive measurement times on (**a**)—day 1, (**b**)—day 2, (**c**)—day 3, and (**d**)—day 4(3′—start day; 3′’—end day).

Figure 5 presents the movement of a scale in the middle cone segment throughout the four-day seed extraction process by showing the location of reference points 1, 2, and 3 at consecutive process times.

The mean opening angle of scales in the middle cone segment increased from 102.08° to 150.03° (by 47.95°, the largest increment) on the first day, from 113.30° to 150.22° (by 36.92°) on the second day, from 111.54° to 149.84° (by 38.3°) on the third day, and from 122.23° to 149.82° (by 27.59°) on the fourth day.

### 3.2. The Cellular Structure of Scales

Larch scales consist of three major cell types:

- Small cells with thick cell walls occur in bundles in the central layer,
- Medium-sized cells with thick walls in the outer and inner epidermal layers,
- Large cells with thin walls and large lumina in the central layer.

**Figure 6:**
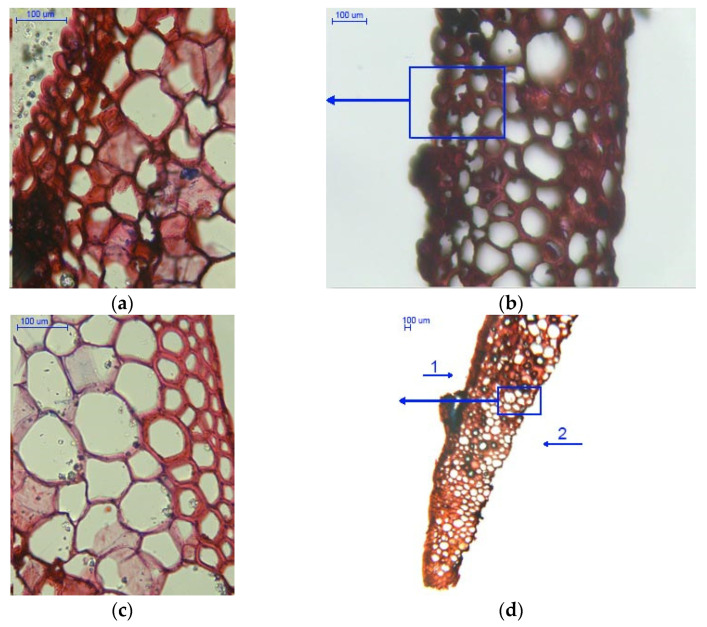
Cross-sections of scales with a moisture content of 5%: (**a**) outer epidermal cell layer (400×); (**b**) scale with visible cell layers (100×); (**c**) scale with visible cell layers on the inner side (400×); and (**d**) scale with visible cell layers in the marginal region (40×), 1—outer side of the scale, and 2—inner side of the scale.

Cross-sections of scales with a moisture content of 5% are shown in Figure 6.

Figure 6a show medium cells of the outer epidermis with a mean cell wall thickness of 2.750 ± 0.530 µm as well as inner epidermal cells with a mean wall thickness of 7.007 ± 1.376 µm. The darker regions in the large cell layer probably represent bundles of cells.

As can be seen from Figure 6b, the epidermal cells on the outer side of the sale are packed more tightly than those on the inner side. Due to loss of water, the cells on the inner side contract, causing scale deflection from the rachis in larch cones (albeit the deflection is less pronounced than in spruce and pine cones).

Figure 6c presents a cross-section involving a scale margin on which glycerin particles penetrated into empty intercellular spaces that emerged as a result of moisture loss during seed extraction. It should also be noted that loss of moisture led to cell deformation.

Figure 6d shows large cells with a cell wall thickness of 3.497 ± 0.946 µm and epidermal cells on the inner side of the scale; their lumina were larger than those on the outer side.

**Figure 7:**
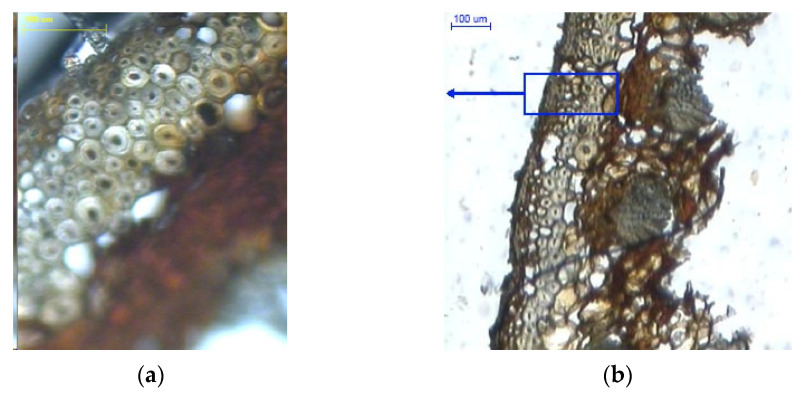

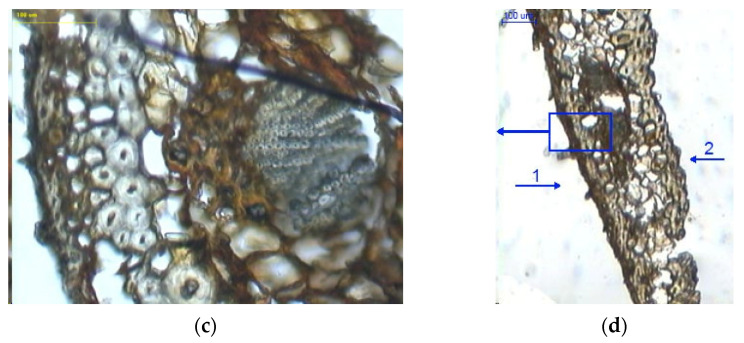
Cross-sections of scales with a moisture content of 20%: (**a**) outer epidermal cell layer (100×); (**b**) scale with visible cell layers on the inner side (100×); (**c**) cell layer in bundles between large cells (100×); (**d**) scale with visible cell layers in the marginal region (40×), 1–outer side of the scale, and 2–inner side of the scale.

Figure 7 presents cross-sections of scales with a moisture content of 20%. Figure 7a shows cross-sections of outer and inner epidermal cells, while large cells and cells in bundles are shown in Figure 7c. Outer and inner epidermal cells had mean wall thicknesses of 3.670 ± 0.561 µm and 9.458 ± 2.335 µm, respectively. The difference in cell wall thickness between the inner and outer scale sides could be the reason why the opening process of larch cones is slower than that of Scots pine and Norway spruce cones.

In Figure 7b, in the middle of the cross-section one can see large cells and cells in bundles with a mean wall thickness of 8.206 ± 1.482 µm and 3.313 ± 0.599 µm, respectively. It was found that the layer of large cells narrows down towards the scale margin (Figure 7d).

A comparison of mean cell wall thickness in scales with a moisture content of 5% and 20% indicates that in medium-sized cells it increased by 25% (outer side) and by approx. 18% (inner side), as compared to approx. 57% for large cells. Analysis of variance revealed significant wall thickness differences between large cells (*p* = 0.00) and medium-sized cells both on the outer side (*p* = 0.00) and inner side of the epidermis (*p* < 0.05) in dry scales (5% moisture) and wet scales (20% moisture).

### 3.3. Results of Structural Examinations of Cone Scales

**Figure 8:**
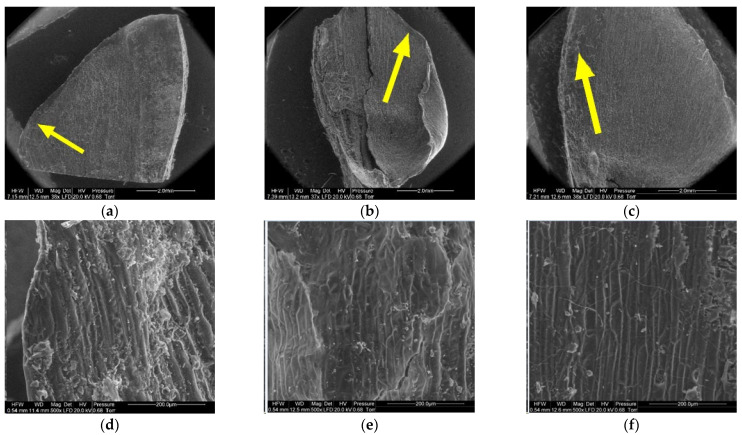
Inner side of larch scale. Scale margin outside of the wing area: (**a**) MC = 20%, zoom 50×; (**b**) MC = 10%, zoom 50×; (**c**) MC = 5%, zoom 50×; (**d**) MC = 20%, zoom 500×; (**e**) MC = 10%, zoom 500×; and (**f**) MC = 5%, zoom 500×.

**Figure 9:**
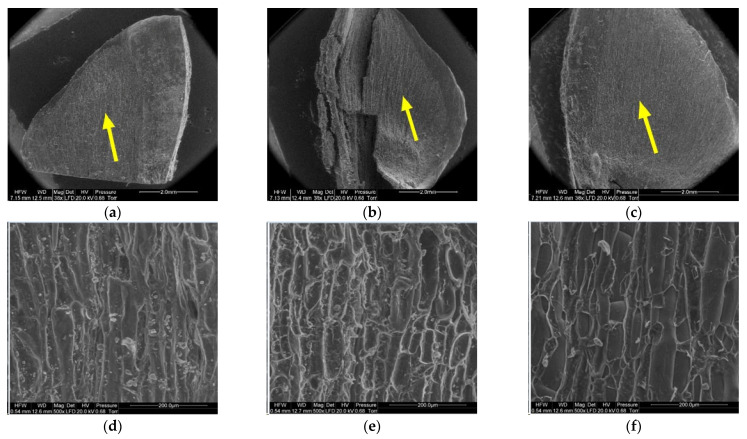
Inner side of larch scale. Wing area: (**a**) MC = 20%, zoom 50×; (**b**) MC = 10%, zoom 50×; (**c**) MC = 5%, zoom 50×; (**d**) MC = 20%, zoom 500×; (**e**) MC = 10%, zoom 500×; and (**f**) MC = 5%, zoom 500×.

**Figure 10:**
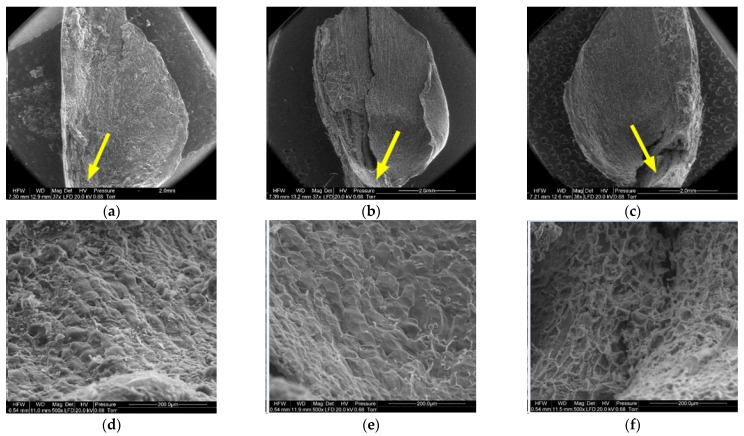
Inner side of larch scale. Seed depression area: (**a**) MC = 20%, zoom 50×; (**b**) MC = 10%, zoom 50×; (**c**) MC = 5%, zoom 50×; (**d**) MC = 20%, zoom 500×; (**e**) MC = 10%, zoom 500×; and (**f**) MC = 5%, zoom 500×.

**Figure 11:**
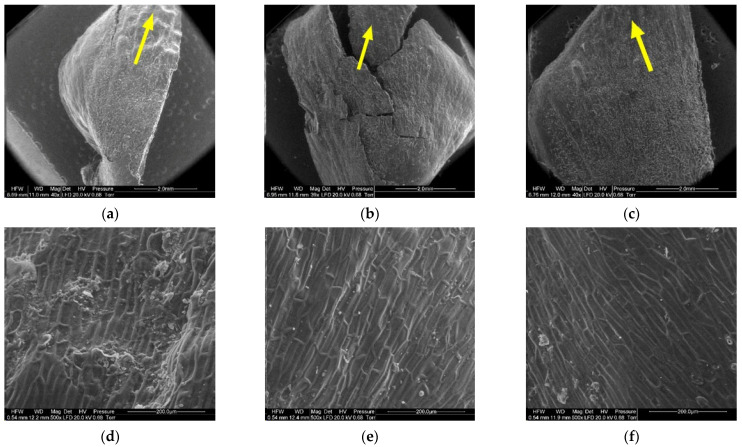
Outer side of larch scale. Distal scale area: (**a**) MC = 20%, zoom 50×; (**b**) MC = 10%, zoom 50×; (**c**) MC = 5%, zoom 50×; (**d**) MC = 20%, zoom 500×; (**e**) MC = 10%, zoom 500×; and (**f**) MC = 5%, zoom 500×.

**Figure 12:**
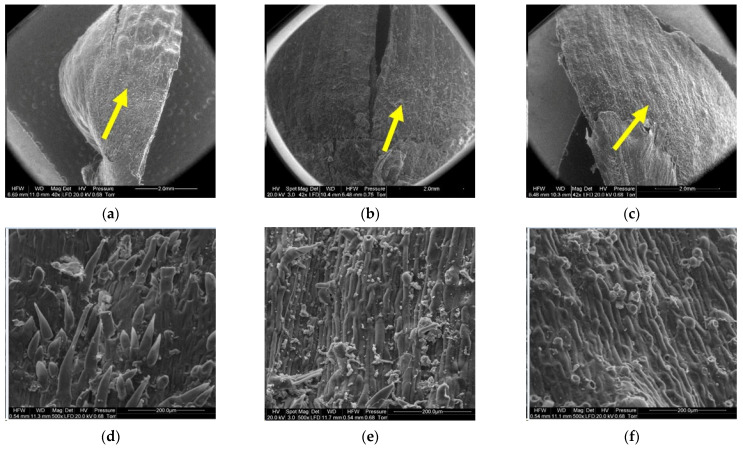
Outer side of larch scale. Middle scale area, adjoin by a lower scale: (**a**) MC = 20%, zoom 50×; (**b**) MC = 10%, zoom 50×; (**c**) MC = 5%, zoom 50×; (**d**) MC = 20%, zoom 500×; (**e**) MC = 10%, zoom 500×; and (**f**) MC = 5%, zoom 500×.

**Figure 13:**
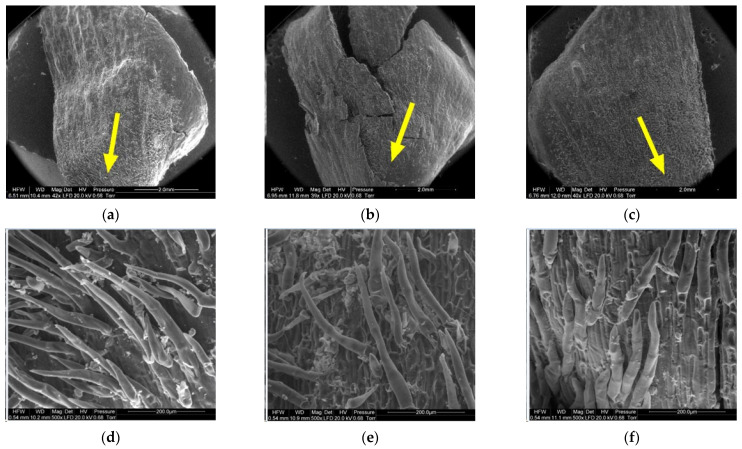
Outer side of larch scale. Proximal scale area: (**a**) MC = 20%, zoom 50×; (**b**) MC = 10%, zoom 50×; (**c**) MC = 5%, zoom 50×; (**d**) MC = 20%, zoom 500×; (**e**) MC = 10%, zoom 500×; and (**f**) MC = 5%, zoom 500×.

Figure 8, Figure 9, Figure 10, Figure 11, Figure 12 and Figure 13 present the results of structural examinations of the inner and outer sides of larch cones with moisture contents of 5%, 10%, and 20%.

On the scale margin, outside of the wing area (1) there are elongated cells differing in their wall thickness. Table 3 shows means with standard deviations as well as minimum and maximum values for the studied cells.

The largest dimensions and wall thicknesses were found for elongated cells in scales with a moisture content of 20% (length of 185.57 µm, width of 17.69 µm, and wall thickness of 7.82 µm). Cells in scales with a moisture content of 10% were much smaller and had thinner walls due to loss of moisture (length of 119.45 µm, width of 15.71 µm, and wall thickness of 5.45 µm). Cells in scales with a moisture content of 5% were slightly smaller than those in scales with a moisture content of 10% (length of 119.35 µm, width of 15.67 µm, and wall thickness of 4.71 µm), with the difference not being statistically significant in the Duncan test. Significant differences were found for the length and width of cells in scales with a moisture content of 20% (*p* < 0.001).

Another region on the inner side of the scale, the wing area (2), featured elongated cells similar to those at the wing margin (1).

Cells in scales with a moisture content of 20% were convex, cylindrical, and overlapping, and had the thickest cell walls (from 4.90 µm to 13.50 µm, with a mean of 9.60 ± 1.44 µm). Their surface featured clusters of particles, probably consisting of resin. Cells in scales with a moisture content of 10% were not convex, having a concave interior, and a wall thickness ranging from 4.60 µm to 8.90 µm (on average 6.50 ± 1.31 µm). Cells in scales with a moisture content of 5% were characterized by thin, damaged walls with a thickness from 3.20 µm to 8.20 µm (on average 5.17 ± 2.09 µm); their surface layer revealed defragmentation.

On the inner side of the proximal part of scales, there were usually two seed nests, but they were not necessarily well-defined or developed on all scales. The seed depression on the inner side of the scale (3) consisted of irregularly-shaped cells. Following seed detachment, the cells were irregular in shape and frayed, as can be seen from the figures presenting this region of the scale (3).

The apical part of the outer side of scales (4) contained closely arranged elongated cells differing in cell wall thickness. The lower the moisture content of the cone, the thinner the cell wall. The wall thickness of cells ranged from 6.20 µm to 10.90 µm, with a mean of 8.02 ± 1.29 µm in scales with a moisture content of 20%; from 5.40 µm to 9.20 µm, with a mean of 6.57 ± 1.08 µm in scales with a moisture content of 10%; and from 4.50 µm to 7.60 µm, with a mean of 6.13 ± 0.81 µm in scales with a moisture content of 5%. Cell wall thickness in scales with a moisture content of 20% was significantly different from that in scales with a moisture content of 5% and 10% (*p* < 0.001 in the Duncan test).

In the case of cones with a moisture content of 20%, the middle part of scales, to which lower scales are adjacent (5), revealed elongated cells with projections in the form of hairs with a mean length of 108.12 ± 54.81 µm and a width at the base of 25.84 ± 3.08 µm. Scales with a moisture content of 10% featured projections with a mean length of 61.78 ± 24.42 µm and a length at the base of 23.20 ± 3.40 µm. No projections were found on scales with a moisture content of 5%; instead, they revealed pores of different diameters—on average 10.76 ± 2.04 µm.

Numerous projections were found on the outer side of the proximal part of scales (6) with all the studied moisture content values. The mean length and width of hair cells on scales with a moisture content of 20% was 265.88 ± 116.72 µm and 28.09 ± 3.39 µm, respectively. Hairs on scales with a moisture content of 10% were 219.99 ± 71.56 µm long and 28.42 ± 5.50 µm wide, while those on scales with a moisture content of 5% were 207.30 ± 48.74 µm long and 31.19 ± 2.82 µm wide. Projections on scales with a moisture content of 5% were the shortest and widest at the base.

## 4. Discussion

As reported by Lin et al. [41] for *Pinus pinaster* cones, the cone opening and closing mechanism can be attributed to the self-bending of their scales, which undergo three states of humidity-driven deformation in terms of Föppl–von Kármán plate theory [42]. Based on three other reports [29,33,35], it may be concluded that it is moisture and the shape and size of cells that trigger opening and closing of cones.

Loss of moisture in the course of drying causes changes in the shape of the treated material [43]. The process of seed extraction involves the contraction of cell walls into the space previously occupied by water and a decrease in the volume of the material [44]. In contrast to other conifer species (spruce or pine), larch cones do not open sufficiently to release seeds freely even in very dry air. The scale structure and opening mechanism for *Pinus radiata* cones were described in detail by Dawson et al. [35], who identified two types of scales growing from the main body of the cone, with the larger ones responding to changes in relative humidity. Therefore, the crucial issue in larch seed extraction is to stimulate cone opening by alternating seed drying and moistening [20], which leads to gradual seed displacement from between the scales.

Larch cones may open to a greater or lesser extent or close depending on air humidity [1], but in the literature there is a dearth of information about the opening of *Larix* cones. In this study, the opening angle of larch scales, defined by the aforementioned three points, increased with decreasing moisture content in the cones.

In the course of four-day seed extraction, the greatest increment in the opening angle was observed on day 1 for scales in the middle cone segment (by approx. 39°), followed by those at the base (by approx. 33°) and at the apex (by approx. 30°). The largest mean increment was found for scales in the middle cone segment. It was calculated that at the end of the extraction process, the scale opening angle ranged from 140° to 145° at a moisture content of 5% to 10%. For larch, the maximum values of the scale opening angle were much higher than those obtained by Bae and Kim [29], who differentiated between the right (120.7°) and left (111.6°) bracts of *Pinus* cones. However, these figures cannot be directly compared due to differences in the angle measurement methodology. The values obtained for larch are close to the maximum angles (approx. 145°) reported by Reyssat and Mahadevan [36] for *Pinus coulteri*.

In view of the finding that neither the scale position in the cone nor the process duration (for various hours on subsequent days) had a significant impact on the scale opening angle, it seems reasonable to shorten the seed extraction process, for example, from four to three days. The yield should be monitored, and if it no longer increases, the extraction process can be terminated.

The initial moisture content in larch scales increased, while the final content decreased, with each day of the process. Cone moistening caused scale closure at the beginning of each day (Figure 3). In the literature there are insufficient data on the number and duration of cone drying and moistening steps needed to maximize seed yield. It is known that an hour-long cone immersion in water is inadvisable due to the swelling of seeds (which must be then promptly sown) [1]. In another study (forthcoming), the authors reported the effects of the number and duration of seed extraction and cone moistening steps on the yield of larch seeds of first class quality. It was found that three 8 h seed extraction steps with two 10 min water immersion treatments in between led to a 59% yield (seeds obtained as compared to the overall number of seeds in the cone).

As reported by, inter alia, Tyszkiewicz [1,20], Bae and Kim [29], Fahn and Werker [30], Bar-On et al. [31], and Reyssat and Mahadevan [36] for cones of *Pinus* or other trees, the structural tissue responds to moisture changes, which trigger the gradual opening of tightly closed cones. The mechanism of cone opening or closing associated with the loss or gain of moisture, respectively, is based on the two-layer structure of cells that transform with changes in moisture content. In the outer layer of the tissue, thick-walled cells respond by expanding in the longitudinal direction when exposed to moisture increase [35,45] and by shrinking in response to to drying, while the simultaneous reaction of cells in the inner layer is feebler.

The microscopic structure of scales revealed cells differing in terms of their wall thickness. Larch scales consist of three types of cells: small, medium-sized, and large. During seed extraction, the cells changed their dimensions, due to which they wrinkled and deflected from the cone rachis [4] to release seeds [37]. This is associated with the close adjoining of epidermal cells on the outer side (Figure 6a), where the cell lumen is much smaller than in the case of inner epidermal cells (Figure 6d). This may be related to the wall thickness of medium-sized cells, which was not affected by loss of moisture to the same extent as the wall thickness of large cells (a decrease of approx. 57%). Furthermore, as reported by Aniszewska [34], between three and five layers of cells may be identified on cross-sections of spruce cones, depending on the scale position. Near the stem, the small, medium-sized, and large cells have diameters of 56.7 µm, 32.3 µm, and 15.3 µm, respectively.

Cells in *Pinus* scales with a moisture content of 20% had thicker cell walls and smaller lumina than those in scales with a lower moisture content. A study on the thickness of cell walls in larch wood reported 20–23 µm for wood samples dried to a moisture content of 5%–15% [46], which is consistent with the results obtained in this paper.

The outer and inner structures of cones scales are different. In the middle segment of the outer surface (5), scales with the highest moisture content revealed short projections, which decreased in length and width with the degree of moisture loss from the cone. In this region (5), scales with the lowest moisture content featured pore-like structures, which probably enabled the elimination of excess water from the cones [4]. The projections were situated on the outer side of scales with all studied levels of moisture content. The proximal part of the scale (6) exhibited hairs, whose length increased with the moisture content of the cone. Hairs (6) on scales with a moisture content of 5% had the greatest width at the base and adhered to the scale surface, while hairs (6) on scales with a moisture content of 20% and 10% formed a bristle. *Pseudotsuga menziesii* and *Abies alba* have scales of a similar structure between seeds [47].

Resin particles were found on scales with the highest moisture content, while scales with lower moisture content values did not reveal such particles; in the latter case they tended to come off, which indicates that the first by-product of seed extraction from conifer cones is dried resin (colophony) [1].

Studies show that the seed extraction process does not have to be conducted over four days since three days with two moistening treatments in between is sufficient. On the last day, the change in the opening angle is lower than that on the preceding days, while moisture content does not decrease below the level obtained on day 3.

## 5. Conclusions

The opening angle of larch scales increased with decreasing moisture content in the cones. The greatest increment in the opening angle was observed on the first day of seed extraction (on average 34° for the three types of scales). The largest mean opening angle increment was found for scales in the middle segments of the studied cones; the largest mean opening angle was 145.99°.

The size and thickness of cell walls in scales is determined by the moisture content of the cones: the higher it is, the thicker the cell walls (up to the fiber saturation point of approx. 30%). Conversely, the lower the moisture content, the larger the cell lumen. The thickest walls were found in the inner epidermal cells (9.458 µm), and the thinnest walls in vascular bundle cells (3.313 µm). In turn, the greatest change in wall thickness was identified in the large cells found in the middle scale segment, with the mean difference between dry and moist states amounting to 4.708 µm. The mean wall thickness of large cells in scales with a 5% moisture content amounted to 42% of that in scales with a 20% moisture content.

The outer and inner scale structures differed depending on moisture content. The greatest differences in the surface structure of scales with 5%, 10%, and 20% moisture contents could be observed on the outer side. On scales with a 20% moisture content, the hair-like cells were elongated and strongly deflected outwards; in contrast, on scales with a 10% moisture content, there were fewer such cells, which exhibited constrictions and leant towards the cone rachis. On scales with a 5% moisture content, the hairs were short and adhered to the outer scale surface. The inner sides of scales with moisture contents of 5%, 10%, and 20% differed significantly at the scale margin outside of the wing area. Resin particles were found on both sides of scales with a 20% moisture content but not on scales with a 5% moisture content.

The results of our investigation of the scale opening kinematics and the cellular structure of larch cones depending on the cone moisture content and the duration and stage of seed extraction may contribute to determining the conditions for the automation of this process.

## Figures and Tables

**Table 1:** Characteristic parameters of the studied cones.

Data	Mean ± SD	Min.	Max.	Range	Coefficient of Variation
Length *h*, mm	31.2 ± 1.0	30.0	33.1	3.1	3.2
Thickness *d*, mm	16.3 ± 0.6	15.5	17.1	1.6	3.7
Initial mass *m*_0_, mm	1.247 ± 0.398	0.725	2.052	1.327	31.9
Mass of dry cone *m_s_*, g	0.946 ± 0.306	0.546	1.568	1.022	32.3
Number of scales *l_w_*, [pcs]	53 ± 5	45	61	16	10

Note: SD is standard deviation.

**Table 2:** Mean moisture content in cones and the corresponding opening angles of scales in the bottom, middle, and top cone segments over four consecutive days of measurement.

Time	Day 1		Day 2		Day 3		Day 4	
[h]	*u*_1_	*α*_1_	*u*_2_	*α*_2_	*u*_3_	*α*_3_	*u*_4_	*α*_4_
Scale from the bottom of the cone segments								
0	0.326 ± 0.016	100.98 ± 10.28	0.346 ± 0.117	104.78 ± 10.50	0.377 ± 0.085	106.79 ± 10.83	0.364 ± 0.106	111.53 ± 9.80
1	0.260 ± 0.016	116.06 ± 13.35	0.286 ± 0.088	117.51 ± 12.59	0.282 ± 0.111	118.70 ± 10.68	0.232 ± 0.091	124.20 ± 10.07
2	0.197 ± 0.015	121.46 ± 12.87	0.242 ± 0.064	122.84 ± 10.72	0.232 ± 0.070	122.91 ± 11.65	0.174 ± 0.069	127.73 ± 10.38
3	0.156 ± 0.010	126.64 ± 11.60	0.135 ± 0.043	129.05 ± 10.30	0.112 ± 0.039	129.45 ± 11.18	0.098 ± 0.042	131.31 ± 10.54
4	0.104 ± 0.014	129.23 ± 11.01	0.103 ± 0.024	131.92 ± 9.52	0.095 ± 0.024	131.29 ± 10.18	0.078 ± 0.023	132.68 ± 10.56
5	0.090 ± 0.008	130.53 ± 10.88	0.091 ± 0.013	132.90 ± 9.68	0.080 ± 0.012	132.55 ± 10.28	0.070 ± 0.012	134.42 ± 10.70
6	0.083 ± 0.004	131.74 ± 10.81	0.081 ± 0.006	133.81 ± 9.70	0.076 ± 0.008	133.40 ± 10.17	0.064 ± 0.008	135.18 ± 10.68
7	0.081 ± 0.004	132.93 ± 11.09	0.078 ± 0.005	134.56 ± 9.89	0.071 ± 0.011	134.51 ± 10.44	0.063 ± 0.008	136.10 ± 10.89
8	0.077 ± 0.004	133.88 ± 10.95	0.075 ± 0.004	135.13 ± 9.79	0.069 ± 0.012	135.42 ± 10.39	0.062 ± 0.008	136.88 ± 10.64
Scale from the middle of the cone segments								
0	0.329 ± 0.018	106.40 ± 9.86	0.387 ± 0.091	108.63 ± 10.72	0.421 ± 0.069	108.37 ± 11.73	0.425 ± 0.113	113.23 ± 11.15
1	0.265 ± 0.019	125.93 ± 9.93	0.306 ± 0.062	124.81 ± 10.47	0.301 ± 0.078	124.52 ± 11.10	0.270 ± 0.083	127.76 ± 11.39
2	0.198 ± 0.017	131.90 ± 9.91	0.251 ± 0.046	129.99 ± 11.11	0.230 ± 0.046	129.85 ± 11.39	0.192 ± 0.059	130.92 ± 11.27
3	0.156 ± 0.013	138.66 ± 10.06	0.135 ± 0.035	137.28 ± 11.53	0.106 ± 0.030	138.02 ± 11.46	0.104 ± 0.034	137.53 ± 11.78
4	0.103 ± 0.013	140.87 ± 10.33	0.101 ± 0.021	139.80 ± 11.52	0.093 ± 0.018	139.66 ± 12.02	0.078 ± 0.018	140.14 ± 11.84
5	0.091 ± 0.010	141.89 ± 10.38	0.089 ± 0.013	140.92 ± 11.38	0.080 ± 0.009	141.07 ± 11.91	0.069 ± 0.010	141.66 ± 11.66
6	0.085 ± 0.008	143.16 ± 10.58	0.081 ± 0.009	142.55 ± 11.55	0.076 ± 0.006	141.96 ± 12.06	0.064 ± 0.007	142.71 ± 11.60
7	0.082 ± 0.008	144.03 ± 10.69	0.078 ± 0.008	143.48 ± 11.56	0.073 ± 0.006	142.88 ± 11.97	0.063 ± 0.007	143.47 ± 11.80
8	0.079 ± 0.008	144.99 ± 10.88	0.076 ± 0.008	144.25 ± 11.53	0.073 ± 0.005	143.84 ± 12.00	0.062 ± 0.007	144.02 ± 11.74
Scale from the top of the cone segments								
0	0.334 ± 0.018	108.97 ± 5.81	0.377 ± 0.113	110.96 ± 6.15	0.416 ± 0.104	111.09 ± 5.97	0.415 ± 0.118	114.65 ± 6.95
1	0.267 ± 0.020	122.41 ± 7.91	0.296 ± 0.079	122.40 ± 8.33	0.291 ± 0.105	122.19 ± 6.76	0.263 ± 0.098	125.28 ± 7.30
2	0.196 ± 0.022	127.59 ± 8.94	0.242 ± 0.060	126.50 ± 9.03	0.225 ± 0.066	128.20 ± 7.33	0.186 ± 0.070	129.93 ± 8.54
3	0.152 ± 0.014	132.98 ± 8.06	0.129 ± 0.043	133.00 ± 8.69	0.102 ± 0.039	133.48 ± 8.01	0.098 ± 0.040	134.17 ± 7.80
4	0.101 ± 0.017	135.03 ± 8.21	0.099 ± 0.025	135.56 ± 8.53	0.091 ± 0.023	135.86 ± 7.17	0.075 ± 0.022	136.16 ± 7.97
5	0.090 ± 0.012	136.71 ± 8.09	0.088 ± 0.015	136.76 ± 8.44	0.078 ± 0.012	136.22 ± 8.23	0.066 ± 0.012	137.83 ± 7.88
6	0.084 ± 0.010	137.81 ± 7.75	0.079 ± 0.010	137.64 ± 8.38	0.074 ± 0.008	137.05 ± 8.23	0.062 ± 0.008	139.09 ± 8.25
7	0.082 ± 0.009	138.67 ± 7.74	0.077 ± 0.009	138.79 ± 8.39	0.070 ± 0.010	138.35 ± 8.14	0.061 ± 0.008	139.89 ± 8.42
8	0.078 ± 0.009	139.14 ± 7.59	0.075 ± 0.008	139.64 ± 8.25	0.069 ± 0.011	139.55 ± 8.17	0.060 ± 0.008	140.65 ± 8.48

Note: *u_1_–u_4_* is mean moisture content ± SD [${\mathrm{kg}}_{\mathrm{water}}\cdot {\mathrm{kg}}_{\mathrm{dry}\;\mathrm{weight}}^{-1}$]; *α_1_–α_4_* is mean angle opening angle ± SD [°].

**Table 3:** Cell sizes at the scale margin outside of the wing area (1) on the inner side of scales with a moisture content of 5%, 10%, and 20%.

	**Moisture Content of Scales**								
**Data**	**20%**			**10%**			**5%**		
	**Mean ± SD**	**Min.**	**Max.**	**Mean ± SD**	**Min.**	**Max.**	**Mean ± SD**	**Min.**	**Max.**
Length [µm]	187.57 ^a^ ± 47.00	105.70	274.40	119.45 ^b^ ± 37.14	95.40	217.80	119.35 ^b^ ± 28.18	92.10	208.60
Width [µm]	17.69 ^a^ ± 3.48	9.80	24.00	15.71 ^a^ ± 2.40	11.40	20.60	15.67 ^a^ ± 3.93	10.70	23.80
Wall thickness [µm]	7.82 ^a^ ± 1.74	5.20	12.10	5.45 ^b^ ± 0.92	4.10	7.00	4.71 ^b^ ± 1.22	2.90	7.00

Note: ^a,b^—homogeneous groups.

## Data Availability

Not applicable.
